# Which incentive package will retain regionalized health personnel in Burkina Faso: a discrete choice experiment

**DOI:** 10.1186/1478-4491-12-S1-S7

**Published:** 2014-05-12

**Authors:** Fadima Yaya Bocoum, Eddine Koné, Seni Kouanda, W Maurice E Yaméogo, Aristide Romaric Bado

**Affiliations:** 1 Département biomédical et santé publique, Institut de Recherche en Science de la Santé, Ouagadougou, Burkina Faso; 2 Kaya Health and Demographic Surveillance System (Kaya HDSS), Burkina Faso; 3 Institut Africain de santé publique (IASP), Ouagadougou, Burkina Faso

**Keywords:** discrete choice experiment, recruitment, retention, health workers, incentive, Burkina Faso, méthode des choix multi-attributs, recrutement, rétention, travailleurs de la santé, mesures incitatives, Burkina Faso

## Abstract

**Background:**

The lack of motivation of health workers to practice in rural areas remains a crucial problem for decision-makers, as it deprives the majority of access to health care. To solve the problem, many countries have implemented health worker retention strategies. However, the development of such strategies requires an understanding of the preferences of health workers. The objective of the study was to identify a package for attracting and retaining health workers in underserved areas.

**Methods:**

A cross sectional study was conducted in three health regions of Burkina Faso in 2012. A discrete choice experiment was used to investigate preferences for incentive packages among health workers recruited under the regionalized policy. In-depth interviews and focus group discussions with health workers currently working in the East and Sahel regions and policy makers, and a literature review on attraction and retention in low income countries, were performed to identify the attributes and levels. These attributes were: the regionalized recruitment policy, health insurance, work equipment, housing, and specific incentive compensation. The final design resulted in 16 choice sets. A multinomial logistic regression was used to determine the influence of socio-demographic characteristics on choice of a given option. A probit logistic regression model was then used to analyze the effect of these difference variables on choice, to identify the incentive package best suited to health workers. In total, questionnaires were administered to 315 regional health workers.

**Results:**

For all participants, choice of package was strongly influenced by length of commitment under the policy and provision of housing. Sex, number of years in profession, and location also influenced the choice of package. Women are twice more likely to choose a package with free housing and the cancellation of the policy.

**Conclusion:**

It is important that governments consider health worker preferences in crafting policies to address attraction and retention in underserved areas. In addition, the methodology of discrete choice experiment has been particularly useful, not only for better understanding the factors explaining the reluctance of health workers to work in underserved areas, but also to provide practical advice to the government, to improve its retention policy.

## Abstract (fr)

**Résumé:**

**Contexte**

Le manque de motivation des ressources humaines en santé à exercer en milieu rural demeure un problème crucial pour les décideurs. Cela prive une majorité de la population de l’accès aux soins. Dans le but de résoudre ce problème, plusieurs stratégies de maintien en poste du personnel de la santé ont été mises en oeuvre dans nombre de pays. Cependant l’élaboration de telles stratégies requiert d’appréhender les préférences des travailleurs de la santé afin que l’ensemble de mesures incitatives soit le plus motivant possible pour les retenir. L’objectif de l’étude était de déterminer les mesures qui permettraient de recruter et de maintenir en poste les travailleurs de la santé dans les régions mal desservies.

**Méthodes**

Une enquête transversale a été réalisée dans trois régions sanitaires du Burkina Faso en 2012. La méthode des choix multi-attributs a été utilisée pour trouver les préférences concernant les paquets de mesures incitatives pour les travailleurs de la santé recrutés dans le cadre de la politique régionalisée. Des entrevues approfondies et des groupes de discussion avec des travailleurs de la santé oeuvrant actuellement dans les régions de l’est et du Sahel et des décideurs ainsi qu’une revue de la littérature internationale sur le recrutement et le maintien en poste de la main-d’oeuvre dans les pays à faible revenu ont été réalisés afin de déterminer les attributs et les niveaux. Ces attributs sont : la politique de recrutement régionalisé, l’assurance-maladie, le matériel de travail, le logement et l’indemnité spécifique de motivation. Le questionnaire définitif comprenait 16 ensembles de choix. Une régression multinomiale logistique a été utilisée pour déterminer l’influence des caractéristiques sociodémographiques sur le choix d’une option donnée. Un modèle de régression logistique probit a ensuite été utilisé pour analyser l’effet de ces variables d’écarts sur le choix d’une option afin d’en dégager l’ensemble de mesures incitatives qui convient le mieux au personnel de santé. Au total, les questionnaires ont été remplis par 315 travailleurs de la santé régionalisés.

**Résultats**

Pour tous les participants, le choix de l’ensemble était très influencé par la durée de l’engagement dans le cadre de la politique de régionalisation et l’offre d’un logement. Le sexe, le nombre d’années dans la profession et le lieu de résidence influaient également sur le choix de l’ensemble de mesures. Les femmes étaient deux fois plus susceptibles de choisir un ensemble incluant le logement gratuit et l’annulation de la politique.

Il est important que les gouvernements considèrent les préférences des travailleurs de la santé lorsqu’ils conçoivent leurs politiques afin de recruter et de maintenir en poste les travailleurs dans les régions mal desservies. En outre la méthode des choix multi-attributs a été particulièrement utile, non seulement pour avoir une meilleure compréhension des facteurs expliquant le refus des travailleurs de la santé à travailler dans les régions mal desservies, mais également pour fournir des conseils judicieux au gouvernement afin d’améliorer sa politique de maintien en poste.

## Background

Most countries, particularly in sub-Saharan African, are experiencing a shortfall in health personnel. The World Health Organization (WHO) estimates that 57 countries, including 36 in sub-Saharan Africa and six in South Asia, have a cumulative shortfall of 4.3 million health care workers. This shortfall is comprised of 2.4 million doctors, nurses, and midwives, with one million on the African continent alone [1]. In addition to this severe shortage of health care workers, there is the issue of their uneven distribution between urban and rural areas, keeping in mind that the majority of the population lives in rural areas [1].

Several countries have implemented recruitment and retention policies in order to keep health care workers in rural or remote areas. In Niger, financial incentives for health personnel working in rural areas has improved the recruitment rate [2], while in Mali the program for the medicalization of rural health areas has retained doctors for four years [3].

In Burkina Faso, a regionalized recruitment policy for nurses, midwives, and auxiliary midwives has been in place since 2003 to correct the uneven distribution of human resources.

Although the factors that affect the motivation of health workers are well documented [4-8], those concerning the preferences of health workers are relatively unknown. Some studies have been conducted, mostly in English-speaking countries in Africa, notably Malawi [9], Ethiopia [10], Uganda [11] and Ghana [12]. All these studies used the discrete choice experiment (DCE) method. DCE can take into consideration the aspirations and preferences of health professionals. It is a good tool for gathering information to guide decision-makers in determining what incentive packages to develop for health human resources and requires a rigorous approach. In French-speaking West Africa, very few studies have used this method to identify the preferences of health care workers.

The aim of our study is to determine an incentive package that can attract and retain health personnel recruited by the regionalization policy in underserved areas.

This study was conducted in three health regions in Burkina Faso, which is located in French-speaking West Africa. The health human resources situation is unsatisfactory. The health worker ratios are among the lowest in the world. In 2011, the ratio of physician was 1:22 017, the ratio of nurse was 1:2 679, and the ratio of midwife was 1:12 754 [13].

The majority of health workers are public sector employees. The government is the main job provider in the health care sector. Among health care professionals, 82% are public sector employees [14]. In terms of financing, the human resources spending in the public expenditures went from 1.8% in 2006 to 2.1% in 2009 [15]. The health worker payment system is aligned with the wage schedule for all civil servants. Hence, health workers do not have special status. In Burkina Faso, the two major urban centres have 54% of the doctors, while they are home to only 10% of the country’s population [7].

To ensure an equal distribution of human resources, the Ministry of Health implemented the regionalized recruitment of health workers in 2003. Within the framework of this regionalized recruitment, qualified officers who were trained at nursing schools are able to join the public service as contract workers through direct competitive selection processes organized by the Ministry of Health, for predetermined health regions. For each health region, a number of positions are identified and a call of interest is released. Before the exam, health workers are registered for a predetermined health region. Thus, after passing the exam, health workers are assigned to predetermined health regions for an unlimited time of commitment. They cannot receive an appointment out of their initial health region. They have some options to change health regions. Firstly, they could change positions with another regionalised health worker from another region. Secondly, after two years, if they are admitted to a professional competition that allows them to change professional category, they can obtain an appointment out of their initial region at the end of their new professional training. The health workers targeted by this regionalized recruitment policy are midwives, nurses, itinerant health officers, and auxiliary midwives.

## Methods

### Type of study

This is a cross-sectional study of health personnel working in the public health sector. Data was collected by six data collectors in February 2012.

### Sampling

The sample size required to conduct a discrete choice experiment varies widely [20]. However, 50 appears to be the minimum size required [9].

The sample was selected using a stratified approach. Three health regions were selected purposively. As such, the Sahel, East, and Boucle du Mouhoun regions were selected. These hard-to-reach regions are often characterized by high maternal mortality ratios, a low ratio of personnel to primary health care centre, and a reputation of being difficult according to health professionals. In addition, these health regions have the highest number of health workers recruited under the regionalized recruitment policy. Each health region is comprised of a number of health districts and a regional hospital. Two health districts were selected in each region based on the number of health officers identified through the census. The two districts with the highest number of officers were selected. In total, six health districts were selected. Each health district covers a number of health centres including several primary health centres and one district hospital. All health centres in the district were selected. In each centre, the questionnaire was administered to all health workers recruited through the regionalization program except those who were absent for sickness or on leave. In total, 315 regional health workers were included.

### Theoretical basis

DCE is a method that has been used in health economics since the 1990s [16]. It was used in health status development studies, determination of patient preferences, and identification of decision-making criteria in medical or political interventions [16]. Currently, it is increasingly being used to determine the preferences of health professionals to motivate them to practice in rural or underserved areas [17].

DCE is an evaluation method that consists of proposing to individuals different hypothetical scenarios typical of the good or service to be evaluated. Faced with a series of hypothetical choices, the individual assigns a level of utility to each scenario presented and chooses the one that would give him maximum utility. This method is founded on the random utility model. According to Lancaster’s consumer theory [18], the satisfaction that an individual (n) derives from the consumption of a good is explained by the combination of good characteristics. When faced with several choices or alternatives, the individual **n** will choose the alternative that provides the highest individual benefit or utility. The individual **n** will choose the alternative **i** from a series of choices **C**, comprised of several other alternatives **j**, if and only if the alternative **i** procures maximum utility **U** (U*_in_* = V*_in_* +Ԑ*_in_*):

U*_in_* > U*_jn_* , 

⇔ (V*_in_* + Ԑ*_in_*) > (V*_jn_* + Ԑ*_jn_*)

⇔ (V*_in_* - V*_jn_* ) > (Ԑ*_jn_*- Ԑ*_in_*)

Given that utility cannot be observed, it is, however, possible to collect the preferences of individuals from among the different alternatives proposed. It is assumed that individuals choose an alternative based on the utility it provides. Consequently, the probability that the individual **n** will choose the alternative **i** from among all (**j**) is expressed as:

P*_n_* (*i*/C) = P*_n_* [(V*_in_* - V*_jn_* ) > (Ԑ*_jn_*- Ԑ*_in_*)], 

⇔ P*_n_* (*i/*C) = P*_n_* [Ԑ*_jn_*< Ԑ*_in_* + (V*_in_* - V*_jn_* )], 

### Discrete choice experiment design

This theory of utility requires a multiple attribute approach that consists of breaking down the examined good into its different components or attributes, to which levels (or states) they are likely to take. A monetary attribute is added, which takes into account the consumer’s budget constraints in the selection process. Breaking down the good into attributes and identifying the levels of attributes results in a process of fractional generation of experiences that consists of combining the attributes to get several scenarios. Each scenario reflects a specific state of the studied good [17].

In this study, we first conducted a qualitative study among health professionals in the target population to identify the different attributes [19]. In-depth interviews and focus group discussions with health workers currently working in the East and Sahel regions and policy makers, and a review of international literature on attraction and retention in low income countries were performed. This study allowed us to identify a total of five attributes, one of which is financial and four of which are non-financial. These attributes are: the regionalized recruitment policy, health insurance, work equipment, housing, and specific incentive compensation. The attribute values or levels were chosen to be realistic (Table 1).

**Table 1:** Final DCE attributes and levels for health workers under regionalised recruitment policy All motivation allowance figures presented in CFA Francs (FCFA); 1 euro = 655 FCFA

Attributes	Levels
Regionalised recruitment policy	• Continuation of the current version of the regionalised recruitment policy (unlimited commitment in one health region) • Cancellation of the measure • Commitment to this health region for 5 years before any new appointment in other region • Commitment to this health region for 10 years

Motivation allowance	• 22 000CFA (33.6 Euro) • 32 000CFA (48.8 Euro) • 42 000CFA (64.1 Euro)

Medical coverage	• 75% reduction in the price of lab exams • 80% reduction in the price of medicines and lab exams • Free medication and lab exams

Work equipment	• Availability of equipment in quantity and good quality • Insufficient in quantity of quality equipment • Availability of equipment in quantity but not a good quality

Housing	• No housing provided and no increase in housing allowance • Free staff housing provided • 25% increase in housing allowance

The experience construction step enabled the creation of a design that combines the different attribute levels, to identify all possible incentive packages that include health workers’ aspirations and preferences.

For the DCE, a labelled choice design with two choices in each choice set was used. The experimental plan was made up of all possible combinations, taking into account the number of attributes and levels of each attribute. The regionalized recruitment policy attribute had four levels and all other attributes had three levels. This specification resulted in a design with 324 (4^1^ x 3^4^) possible combinations. As the possible number of combinations was too high to reasonably conduct a study, a fractional or full factorial experimental plan was generated. The Hahn and Shapiro catalogue was used to select combinations for an orthogonal main effects design, and to organize the selected profiles into the most D-efficient choice design, given our design parameters. The final design was resulted in a 16 choice sets. Respondents were then asked to select their preferred option from each of 16 choice sets (15 random and 1 fixed) or could decide to stay in their current job. They were asked to give the reason for the opt-out option and for how long the chosen option could motivate them in their current position.

The survey questionnaire included three sections. In the first section, an information notice on the study, and the meaning of each attribute and levels was presented. In the second section, information was collected on respondents’ socio demographic and professional characteristics including age, sex, ethnicity, religion, marital status, number of children, current professional title and qualifications, and years worked in the health sector. The third section presented the DCE choice sets.

The full questionnaire was pre-tested among health professionals in the health district of Kaya. Minor corrections were made after the pre-test and before data collection. The questionnaire was administered to participants by six data collectors. Each participant was asked to place him/herself in a hypothetical situation, the implementation of a new policy with an incentive package, before presenting the different choice sets.

### Data analysis

The data entry was performed using CSPro software. The data was analyzed using Stata 12 software. A descriptive analysis allowed us to select the options that recorded the highest percentages of agreement by region and by professional category; these options were then selected to conduct multinomial logistic regression to determine the influence of socio-demographic characteristics on the choice of a given option. We also calculated the difference between the level of attributes according to each alternative, and this was done based on dummy variables. A probit logistic regression model was then used to analyze the effect of these difference variables on the choice of an option in order to identify the incentive package best suited to health workers.

### Ethical considerations

This study is a sub-component of a larger study on a recruitment policy for human resources for health in Burkina Faso, for which ethical clearance was obtained from the Ethics Committee for Health Research in Burkina Faso. Respondents participated on a voluntary basis and could withdraw from the study at any time. Informed consent was obtained from all participants and signed consent forms were obtained prior to the interviews.

## Results

### Socio-demographic characteristics of respondents

The socio-demographic characteristics are presented in Table 2. Among respondents, auxiliary midwives were the most represented (30%), followed by licensed nurses (28%) and state-registered nurses (20%). There were more women than men. Furthermore, the respondents had an average age of 32 years (± 5.3) and average seniority in their current position of four years (±3 years). The majority lived in a rural area. Among the respondents, 44% were in a formal union, 29% were single, and 25% were in a common-law union (Table 2).

**Table 2:** Socio-demographic characteristics of respondents

	N	%
Place of residence		

Urban Rural	133 196	40.4 59.6

Marital status		

Single	94	28.6
Divorced/Separated	7	2.1
Common-law union	82	24.9
Formal union	146	44.4

Professional category		

Itinerant health officers	54	16.4
Birth attendant	98	29.8
Licensed nurse	92	28.0
State midwife	18	5.5
State-registered nurse	67	20.4

Gender		

Male	99	30.1
Female	230	69.9

### Incentive packages chosen

Of the 16 options, in general, options 2, 3, and 5 had the highest acceptance in all health regions, with an average of over 80% of the choices (Table 3). In the East and Boucle de Mouhoun regions, option 5 was the preferred option, while in the Sahel they were options 2 and 3. An analysis by professional category shows that, among field health officers, options 3 and 13 were the most preferred, among auxiliary midwives, options 3, 5, and 2 were preferred, and among licensed nurses, options 5, 2, and 3 were preferred. Among midwives and state-registered nurses, the most preferred options are identical to those identified with highest acceptance. These selected options might motivate respondents to stay in their current area of work for an average of three years (±2).

**Table 3:** Presentation of the content of the most selected options

OPTION 2	OPTION 3	OPTION 5
Regionalization policy: Cancellation of the policy	Regionalization policy: Cancellation of the policy	Regionalization policy: Cancellation of the policy

Motivation allowance: 22 000 CFA (33.6 Euro)	Motivation allowance: 32 000 CFA (48.8 Euro)	Motivation allowance: 32 000 CFA (64.1 Euro)

Medical coverage: 80% reduction in the price of medication and lab exams	Medical coverage: 75% reduction in the price of lab exams	Medical coverage: Free medication and lab exams

Work equipment: Insufficient quantity of quality equipment	Work equipment: Insufficient quantity of quality equipment	Work equipment: Available in quantity and quality

Housing: Provision of staff housing	Housing: 25% increase of housing allowance	Housing: Provision of staff housing

The preferred options of health professionals all include the cancellation of the policy and availability of quality work equipment. Options 2 and 5 also propose the provision of staff housing.

### Socio-demographic factors associated with the choice of incentive packages

Options 2, 3, and 5 are the options that the majority of health professionals preferred. Hence, a multinomial model was applied for each of the three options to determine the predictive factors of the choice of options. Socio-demographic variables related to gender, professional category, seniority, age, region, and marital status, that might explain the choice of a given option, were introduced into each model. The table presents the results of the model’s estimate for each option. The professional category, age, and marital status are not significantly associated with the choice of option (Table 4).

**Table 4:** Estimation model of the socio-demographic factors associated with the choice of options

	Option 2				Option 3				Option 5			
	**OR**	**95% CI**		**p-value significant**	**OR**	**95% CI**		**p-value significant**	**OR**	**95% CI**		**p-value significant**
												
Professional category	1.054	0.938	1.184		1.034	0.921	1.161		1.033	0.921	1.160	
Seniority	1.221	1.104	1.350	***	1.203	1.088	1.329	***	1.198	1.084	1.324	***
Age	0.850	0.711	1.018		0.862	0.721	1.031		0.853	0.714	1.019	
Region	2.369	1.889	2.972	***	2.298	1.835	2.878	***	2.177	1.743	2.719	***
Marital status	0.960	0.720	1.278		0.951	0.714	1.265		0.930	0.610	1.237	
Gender	1.784	1.275	2.497	***	1.725	1.235	2.408	***	1.582	1.138	2.197	**
												
**_cons**	**1.023**	**0.007**	**0.072**	***	**0.029**	**0.009**	**0.089**	***	**0.041**	**0.014**	**0.125**	***

Multinomial logistic regression, OR: odds ratio, CI: confidence interval, **: p< 0.05, ***: p< 0.001

Data source: survey conducted in February 2012

For all options, the models include the significant associated factors of seniority, region, and gender. For seniority, having three or more years of professional experience made respondents more likely to choose option 2. For options 3 and 5, experience is significantly associated with this choice. Region is also determinative in the choice of option. Practicing in the Sahel is significantly associated with the choice of all options (options 2, 3, and 5). Women are twice as likely to choose options 2, 3, and 5 (Table 5).

**Table 5:** Estimation model of the modalities associated with the choice of option

	Option 2				Option 3				Option 5			
	**OR**	**95% CI**		**p-value significant**	**OR**	**[95% CI]**		**p-value significant**	**OR**	**[95% CI]**		**p-value significant**
Seniority												
**1 yr**	**1**											
2 yrs	1.491	0.849	2.619		1.279	0.737	2.221		1.186	0.685	2.054	
3 yrs	5.677	3.080	10.462	***	5.164	2.846	9.369	***	4.972	2.758	8.964	***
4 yrs	1.895	1.110	3.233	**	1.578	0.935	2.662		1.499	0.894	2.514	
5 yrs	6.302	3.537	11.231	***	5.524	3.139	9.721	***	5.379	3.072	9.417	***
6 yrs and more	1.837	1.023	3.299	**	1.649	0.933	2.915		1.496	0.849	2.639	
*Region*												
**East**	**1**											
Mouhoun	1.145	0.756	1.735		1.079	0.718	1.624		1.066	0.712	1.595	
Sahel	5.778	3.557	9.385	***	5.352	3.317	8.637	***	4.853	3.017	7.808	***
*Gender*												
**Male**	**1**											
Female	1.910	1.347	2.710	***	1.878	1.326	2.660	***	1.700	1.206	2.396	**
												
**_cons**	**0.091**	**0.049**	**0.167**	***	**0.110**	**0.061**	**0.200**	***	**0.130**	**0.073**	**0.233**	***

Multinomial logistic regression, OR: odds ratio, CI: confidence interval, **: p< 0.05, ***: p< 0.001

Data source: survey conducted in February 2012

### Attributes associated with the choice of incentive package

Table 6 presents the attributes that have an influence on the choice of option. The estimate is significant overall (pseudo R^2^ = 0.1636). Only the attributes related to the regionalization policy and housing are significantly associated with the preferences of health workers. The results for the regionalization policy show that implementation of a measure to establish any kind of term of practice in a region is not preferred by health professionals. The housing results show that the options containing the provision of staff housing for health workers are preferred by health officers. In summary, a health officer would be willing to stay in a rural area if the regionalization policy were cancelled and if decent housing was provided. These results are corroborated by the respondents’ preferred choice of options 2, 3, and 5, in which the regionalization policy is cancelled.

**Table 6:** Influence of the difference variables on the choice of majority options

Variables	Coefficient		[95% CI]	
dpolit	-0.603	***	-0.662	-0.543
dindem	0.002		-0.007	0.012
dcouv	0.026		-0.065	0.117
dmatl	0.027		-0.063	0.118
dlogmt	0.162	***	0.086	0.237
_cons	0.255		0.046	0.464

Probit logit regression, Log likelihood = -1184.919, Pseudo R^2^ = 0.164, CI: confidence interval, dpolit: log of variable regionalised recruitment policy, dindem: log of variable Motivation allowance, dcouv: log of variable Medical coverage, dmatl: log of variable Work equipment, dlogmt: log of variable Housing, ***: p<0.001

Data source: survey conducted in February 2012.

## Discussion

The lack of motivation of health workers to practice in rural areas remains a crucial problem for decision-makers, as it deprives a majority of the population of access to health care. To solve the problem, a number of countries have implemented several health worker retention strategies. However, the development of such strategies requires an understanding of the preferences of health workers so that the incentive package can be as motivating as possible in order to retain them. More and more studies are using the Discrete Choice Experiment method to determine the preferences of health professionals. However, the majority of these studies were conducted in English-speaking countries, and among students.

Regarding the identification of the attributes, salary increment was not identified as an attribute during the qualitative study, as it was in many studies. According to interviewed health workers, it was not realistic to suggest that. In fact, salary in Burkina Faso is based on the same scale for all civil servants, which could explain why they did not suggest a salary increment, since change in salary does not only depend on the Ministry of Health.

The results of our study show that the majority of health officers choose packages that provide for the cancellation of the regionalized recruitment policy, the availability of work equipment, and the provision of housing.

Housing is an important factor in health worker retention in rural areas. These results are corroborated by many studies [9,21,22] that have shown the importance of housing in the motivation to relocate.

No less important, the availability of quality work equipment is a factor often cited as motivation for health workers. Retention strategies that include quality medical equipment have a greater influence on workers’ motivation to provide quality care [10,12,23].

The choices of these incentive packages can be explained by the socio-demographic characteristics of seniority, region of practice, and gender. The effects of seniority can be explained by the fact that the more experience health officers have in their field, the more familiar they are with the realities and their professional prospects. Likewise, the contextual factor is determinative in the choice of packages. The contextual realities vary from one region to another, as the content of a package brings satisfaction depending on the environment in which the health officer resides. Women are twice as likely to choose packages where the regionalization policy is revoked. This choice can be explained in part by family reunification, which is an important motivating factor for women. Cancellation of the policy implies mobility, which will allow them to be reunited with their partners, children and other family. These results are supported by studies that have shown that due to family considerations, women are less motivated than men to practice in rural areas [24,25]. In contrast, other studies conducted in Kenya, South Africa, and Uganda [11,26] found no significant association between the choice of package and the above mentioned socio-demographic characteristics.

Cancellation of the regionalization policy is a preference expressed by health officers after a few years of practice. The reason for this preference may be that initially, the health officer is seeking employment and less responsibility. Once the job has been acquired and the health officer is in contact with other officers who are not subject to the policy, the officer’s aspirations change. Family responsibilities, professional opportunities, as well as community integration, contribute to changing the health officer’s motivation to remain in a rural area.

The period during which incentive packages are motivational is also important. In Mali, the medicalization policy retained doctors in rural areas for four years [3]. The duration of motivation forces a review of retention strategies in order to adapt the content to the changes that will occur in the motivation factors of health officers.

One of the objectives of the study was also to inform policy makers. A few weeks after the survey, health workers were on strike and one of their claims was the cancellation of the unlimited time of commitment of the policy. At the end, the Ministry of Health decided to limit the time of commitment to five years, which was one level in our policy attributes table. Currently, after five years the health worker who is recruited under the regionalised policy could have an appointment in any other region of the country.

Our study has its limits. The limits of the DCE method have been extensively documented [27]. Our current analysis presents results for a heterogeneous group of health workers. Preferences are shaped by context but also by professional category. A more detailed analysis would be useful in determining a package adapted to each region and body. Nonetheless, this does not affect the validity of our results, which can inform decision-makers regarding a general health worker retention strategy.

## Conclusions

Our study shows that the cancellation of the policy and the provision of housing are important determinants in the choice of health officers to practice in rural areas. This could be a first step in the development of an incentive package. Our results also show that one uniform retention policy for health officers is not appropriate. In summary, the DCE method can facilitate the definition of incentive packages to help decision-makers develop interventions adapted to local conditions.

## List of abbreviations

DCE: Discrete choice experiment; DFATD: Foreign Affairs, Trade and Development Canada; GHRI: Global Health Research Initiative; IDRC: International Development Research Centre; WHO: World Health Organization

## Competing interests

The authors declare that they have no competing interests.

## Authors' contributions

FYB, EK, and SK collaborated in the writing of the manuscript. FYB, EK, MWEY, ARB, and SK were involved in the design of the survey. FYB, EK, and MWEY were involved in the conducting of the survey. EK and ARB performed the statistical analyses. All authors revised the manuscript before submission.

## Declarations

The publication costs associated with this article are funded by Foreign Affairs, Trade and Development Canada and the International Development Research Centre through the Global Health Research Initiative.

This article has been published as part of *Human Resources for Health* Volume 12 Supplement 1, 2014: Addressing the human resources for health crisis through task-shifting and retention: results from the Africa Health Systems Initiative's research component. The full contents of the supplement are available online at http://www.human-resources-health.com/supplements/12/S1
